# Public perception of socially assistive robots for healthcare in the EU: A large-scale survey

**DOI:** 10.1016/j.chbr.2024.100465

**Published:** 2024-08

**Authors:** Laura Aymerich-Franch, Emilia Gómez

**Affiliations:** a Universitat Pompeu Fabra, Spain; b European Commission, Joint Research Centre, Spain

## Abstract

This paper presents the results of a large-scale survey (n = 1092) that explored the attitudes and opinions of European Citizens regarding the adoption of socially assistive robots (SARs) for healthcare in the EU. We examined which functions citizens would support and which they consider a threat to trustworthy SARs. We additionally explored the relationships between the perceived vulnerability of the care recipient and acceptance, between attitudes towards robots and gender, age, religious beliefs, and previous experience interacting with SARs, and whether the degree of responsibility taken in performing a role affects acceptance. We also compared attitudes towards robots across European regions. The functions most negatively rated were triage and banning entrance. Privacy raised particular concern. We also found an inverse correlation between the perceived vulnerability of care recipients and acceptance. Additionally, we found a positive relationship between religious beliefs and fear of robots, a positive relationship between previous robot experience and attitudes towards them, and that females have less positive attitudes towards robots than males. Also, the degree of responsibility in a role determined acceptance. Involving citizens in the decisions concerning SARs deployment is important to build a society that people feel is fair in terms of robot coexistence. The results of the survey intend to provide evidence-based support to policies in this area.

## 1 Introduction

Socially assistive robots (SARs) are those specifically designed to assist humans through social interactions (Feil-Seifer & Matarić, 2005). These robots are progressively being deployed in real scenarios (Aymerich-Franch & Ferrer, 2023) and everything indicates that people will have to coexist with these machines in the near future. Faced with this prospect, how can we build a society that citizens consider trustworthy regarding coexistence with robots? What roles and functions do robots need to have (or not have) so that they are not perceived as a threat? How to ensure that social robots can be trusted?

The concept of *trustworthy artificial intelligence* (AI) was introduced by the European Commission's High-Level Expert Group (2019) in their ethical guidelines (Smuha, 2019). It is conceived as a set of seven requirements that AI systems should fulfill in order to be considered trustworthy. These include: (1) human agency and oversight, ensuring proper human supervision and control over AI systems; (2) technical robustness and safety, addressing the need for secure and resilient systems against attacks; (3) privacy and data governance, considering respect for personal data; (4) transparency, so that AI systems are documented with respect to their functioning, performance, and limitations; (5) diversity, non-discrimination and fairness, ensuring that AI systems work well for everyone and are developed in an inclusive way; (6) societal and environmental well-being, which deals with the need to ensure the benefit of the systems in terms of society and environment; (7) accountability, that ensures responsibility mechanisms, auditability and redress. However, even if AI system developers may ensure compliance with these seven requirements, what is the user perspective of trustworthiness and how does it depend on the context of use?

Approaching this matter from a bottom-up, citizen-centric perspective that directly involves citizens is then critical to find an answer. Listening to the requirements from citizens regarding robot deployment in Europe can help support policies aimed at building a society that people feel is trustworthy in terms of robot coexistence.

In response to this need, we conducted a large-scale survey among European citizens to inquire about their opinions regarding the adoption of socially assistive robots (SARs) for healthcare in the European Union. In particular, we explored the following research questions regarding functions and their relationship to trustworthiness.

RQ_1_. What functions performed by SARs for healthcare do European citizens consider should be supported and promoted in an ideal European society?
RQ_2_. What functions performed by SARs for healthcare do European citizens consider entailing a potential threat to trustworthy social robots?
  In the human-robot interaction literature, a wide range of factors has been discussed as potentially affecting trustworthiness, perceptions, acceptance, and fear of robots. These include, among many others, care recipients' vulnerability (Liu et al., 2021), people's roles in the healthcare system (Broadbent et al., 2009), religious beliefs (MacDorman et al., 2009), and previous technological experience (Heerink, 2011). We additionally explored the interrelations with and effects of some of these factors on willingness to promote social robots for healthcare and attitudes towards robots. In particular:
RQ_3_. Is there a relationship between the perceived vulnerability of the care recipient and willingness to promote social robots for healthcare?
RQ_4_. Does the degree of responsibility in the role (a robot performing a role in healthcare vs. a robot assisting the human to perform a role) significantly affect the willingness to promote social robots for healthcare?
RQ_5_. Is there a relationship between religious beliefs and fear and attitudes toward robots?
RQ_6_. Is there a relationship between previous experience interacting with social robots and attitudes toward robots?

Finally, we also examined differences in attitudes towards robots by the basic demographics of gender and age, as well as across European regions (Northern-Western, Southern, and Eastern).

In order to conduct the survey, we elaborated a list of 26 functions that SARs for healthcare develop in real settings at present based on the classifications of functions provided by Aymerich-Franch and Ferrer (Aymerich-Franch and Ferrer, 2022, 2023), which participants evaluated in the survey. The functions were related to entertainment and edutainment, companionship, telepresence, providing information, monitoring, rehabilitation and physical exercise, testing and pre-diagnosis, delivery, patient registration, protective measure enforcement and patrolling, patient simulator, providing indications, medication and well-being adherence, translation, psychological support, and disinfection. The full list of functions is provided in the Supplementary Information (SI).

## 2 Method

### 2.1 Ethics declaration

The research protocols were approved by the Joint Research Centre - JRC research Ethics board. All methods were carried out in accordance with relevant guidelines and regulations. Informed consent was obtained from all participants. The participants gave their consent through the EU platform at the start of the survey.

### 2.2 Contents of the survey

The survey contained the following blocks.

**Fig. 1:**
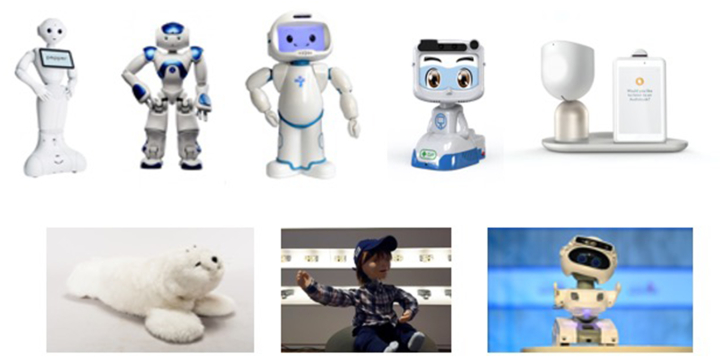
Social robots introduced in the survey. Credits, from left to right, and top to bottom: Pepper: Softbank Robotics Europe, Pepper the Robot, CC BY-SA 4.0; Nao: Softbank Robotics Europe, NAO Evolution, Size, CC BY-SA 4.0; QTrobot: QTrobot, Qtrobot, CC BY-SA 4.0; DinsowMini; Jahjajachay, Dinsow mini, CC BY-SA 4.0; ElliQ: Laranorman, "ELLI 3 RGB.jpg”, CC BY-SA 4.0; Paro, Tekniska museet/Peter Häll, Robotsälen Paro TEKS0057912, CC BY-SA 4.0; Kaspar: Matt Brown from London, England derivative work: M1llx, "Science Museum - Robots - Robot for autistic children (32781592346)--modfied.jpg”, CC BY 2.0; Misty: Web Summit, "071119SMcC0167”, CC BY 2.0.
● Demographics, including country of residence, nationality, gender, age, occupation, work experience in healthcare, size of town, level of studies, marital status, household composition, difficulty paying bills, perceived social class, and religious beliefs (some questions based on (European Commission, 2012))
● Relationship with robots, attitudes towards robots (European Commission, 2012), and fear of robots (some items based on (Liang & Lee, 2017))
● Functions: block of questions to evaluate acceptance towards the different functions carried out by SARs for healthcare identified by (Aymerich-Franch and Ferrer, 2022, 2023)
● Introduction to the 6 dimensions of trustworthy social robots: human autonomy; privacy; safety; fairness, diversity, and non-discrimination; societal well-being; and accountability (Smuha, 2019).
● Threats to trustworthy social robots: block of questions to evaluate the perceived threat to each of the six dimensions of trustworthy social robots for each of the functions identified by (Aymerich-Franch and Ferrer, 2022, 2023)
● Care recipient: block of questions to evaluate the acceptance of SARs depending on the characteristics of the care recipient (e.g., children, older adults …) and the perceived vulnerability of each group of care recipients.
● Role and responsibility: block of questions to evaluate the acceptance of SARs in different roles and also depending on the degree of responsibility in the role (e.g., assist a doctor vs. be the doctor).

● Consent form
● Introduction to the aim of the study
● Introduction to SARs, with eight different pictures of social robots (Fig. 1)

We additionally added four attention check questions among the previous questions (e.g., “I am paying attention to the survey, select “not at all”). The full list of functions is provided in the Supplementary Information (SI).

### 2.3 Platform

The survey was built using the platform EU survey, the European Commission's official survey management tool (https://ec.europa.eu/eusurvey/home/about). Participants were recruited via the recruitment platform Prolific and redirected to EU survey, where they completed the survey. Prolific offers the option to select certain characteristics of the sample. The survey was open to participants aged 18 or older who were fluent in English and were residents in any of the 28 countries of the European Union. We established a balanced criteria for gender. We estimated a time to complete the survey of 35 min and set a compensation of 4.18 EUR. This project was subject to an ethical and data protection review, e.g. the participants signed a consent form online, before starting the survey in EU survey. We conducted the survey between March and July 2022. We analyzed the data using the statistical software SPSS.

## 3 Results

A total of 1127 European citizens completed the survey. On average, participants took 23.6 min to complete the study. Twenty-seven participants were discarded for failing two or more attention checks, and data from eight participants was lost, resulting in a final sample of 1092 responses.

Of the final sample, 529 participants (48.4%) self-identified as female, 541 (49.5%) as male, and 22 (2%) had other gender definitions. The mean age was 27.9 years (SD = 8.37). The age distribution was as follows: 489 participants (44.8%) were 18–24 years old, 411 (37.6%) were 25–34, 133 (12.2%) were 35–44, 42 (3.8%) were 45–54, 16 (1.5%) were 55–65, and one participant was 74 years old.

Participants resided in 20 different EC countries and represented 26 different EC nationalities, with two participants residing in the EC but reporting other nationalities (see Tables 1A and 2A - SI). Among them, 641 lived in cities, 301 lived in towns, and 150 lived in villages or rural areas.

Regarding education, 368 participants had bachelor's degrees or equivalent, 247 had master's degrees or equivalent, 236 had attended some college but did not earn a degree, 190 had a high school degree, 24 had a PhD, 14 had a professional degree, and 13 had less than a high school diploma. In terms of occupation, 413 participants were students, 406 were employed, 100 were unemployed, 74 were both students and employed, 64 were self-employed, 6 were retired, and the rest reported other situations.

Regarding work in the healthcare sector, most participants (959) had never worked in healthcare, 76 had worked in healthcare in the past, and 57 were currently working in the healthcare sector. Of the participants, 392 had a background in Computer Science, Engineering, or Robotics, while 700 did not. Concerning programming skills, 462 had basic programming skills, 410 had none, 157 had medium-level skills, and 63 had advanced skills. Regarding their interest in scientific discoveries and technological developments, 576 were moderately interested, 468 were very interested, and 48 were not interested.

With respect to marital status, 571 participants were single, 372 were in a domestic partnership or had a partner, 109 were married, 15 were divorced or separated, 2 were widowed, and 23 reported other situations. In terms of household composition, 482 participants were living with their parent(s), 220 were living with their partner, 157 were living alone, 115 were living with housemates, 89 were living with their partner and child(ren), 13 were living with their child(ren), and 16 reported other situations. This distribution aligns with the age distribution of the respondents.

Regarding socioeconomic status, 558 participants identified their household as middle class, 379 as lower middle class, 103 as upper middle class, 48 as lower class, and 4 as higher class. When asked, “During the last twelve months, would you say you had difficulties paying your bills at the end of the month?”, 534 participants answered never, 276 answered almost never, 233 answered from time to time, and 49 answered most of the time.

With respect to familiarity with robots, 536 participants had previously used robots at home (e.g., robotic vacuum cleaners), while the rest had not; 208 had used robots at work (e.g., industrial robots), while the rest had not; and 491 had used robots in other places (e.g., airports or malls), while the rest had not. Specifically concerning social robots, 711 had never interacted with social robots, 361 had occasionally interacted with social robots, and 20 had ample experience interacting with social robots.

Finally, regarding religious beliefs, 727 participants were not religious, 251 were slightly religious, 89 were moderately religious, and 25 were very religious. When asked about their religious affiliation, 529 participants identified as Christian, 493 as having no religious affiliation, 24 as Muslim, 8 as Buddhist, 5 as Jewish, 4 as Hindu, and 29 as belonging to other religions.

### 3.1 Attitudes towards robots and fear of robots

The first set of questions examined general attitudes towards robots and robots in healthcare, as well as fear of robots.

**Fig. 2:**
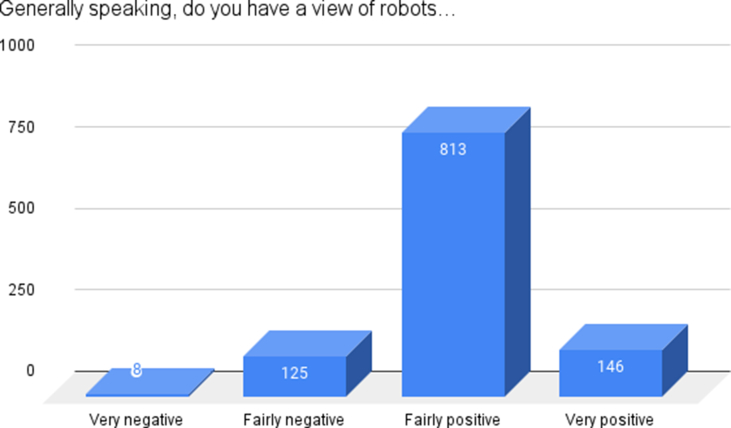
Overall view of robots.

The majority of participants (72.7%, n = 813) reported having an overall fairly positive view of robots (Fig. 2).

**Fig. 3:**
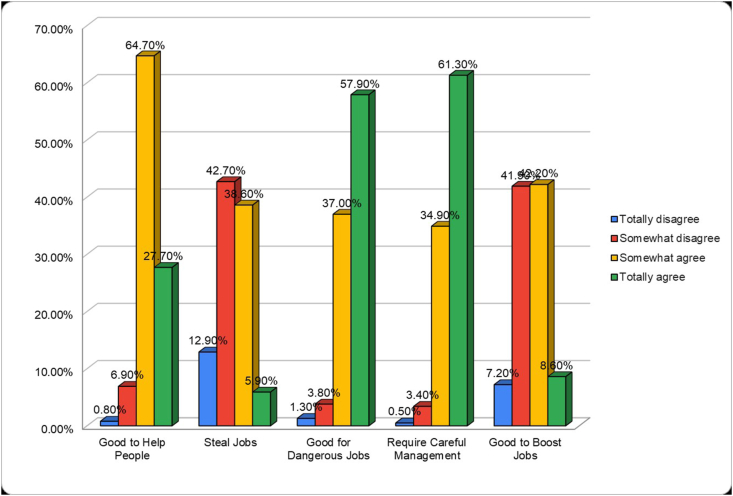
General attitude towards robots.

The questionnaire evaluating general attitudes towards robots included five statements. The majority of the sample somewhat agreed (63.1%, n = 706) with the statement “robots are a good thing for society because they help people.” Opinions were split on whether robots steal people's jobs, with 37.7% (n = 421) somewhat agreeing and 41.7% (n = 466) somewhat disagreeing. Most participants totally agreed (56.5%, n = 632) that robots are necessary as they can perform jobs that are too hard or too dangerous for people. A significant portion (59.8%, n = 669) totally agreed that robots are a form of technology that requires careful management. Opinions were also divided on whether the widespread use of robots can boost job opportunities, with 41.2% (n = 461) somewhat agreeing and 41% (n = 458) somewhat disagreeing. The full results of the general attitudes towards robots questionnaire are shown in Fig. 3.

**Fig. 4:**
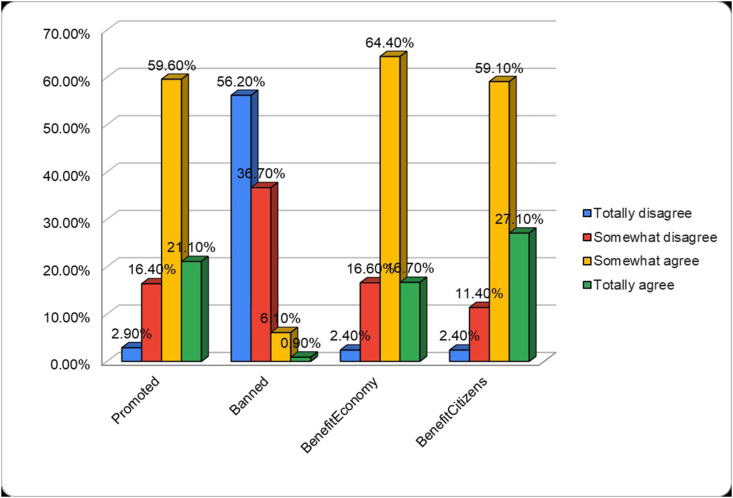
Attitude towards robots for healthcare.

The questionnaire evaluating attitudes towards robots in healthcare contained four statements. The majority of participants somewhat agreed (58.2%, n = 651) that robots for healthcare should be promoted. A majority (54.9%, n = 614) disagreed that robots for healthcare should be banned. Additionally, 62.9% (n = 703) somewhat agreed that robots in healthcare can be beneficial for the economy, and 57.7% (n = 645) somewhat agreed that they can be beneficial for citizens. The full results of the attitudes towards robots in healthcare questionnaire are shown in Fig. 4.

Fear of robots was assessed using a 12-item scale with a 5-point range (1 = not at all, 5 = very strongly) and showed good reliability (Cronbach's α = 0.89). On average, participants reported a moderate fear of robots (M = 2.22, SD = 0.79).

### 3.2 Willingness to promote the different functions carried out by SARs for healthcare

Participants were asked to rate the following statement on a 4-point scale (1 = totally disagree, 2 = somewhat disagree, 3 = somewhat agree, 4 = totally agree) for each of the functions (see SI): “In the ideal European society that you envision for the near future, European policymakers support and promote the development and deployment of social robots that perform the following functions … "

The functions that were rated most negatively (M < 3) were measuring vital signs so that the robots themselves can provide pre-diagnoses or do the triage (i.e., prioritize patients according to the degree of urgency) based on the symptoms and banning entrance to a building to people that present a threat to public health (e.g., have a fever) or do not comply with the rules (e.g., mandatory face mask).

Offering motivational conversation to patients of a hospital, expressing and interpreting emotions to communicate, providing touch interaction for emotional support, providing personalized information such as informing about the characteristics of a particular medical condition, symptoms, and potential treatments to patients in hospitals, and collecting personal information of medical symptoms presented by patients on arrival at the hospital or medical center also obtained a negative evaluation (M < 3).

The remaining functions were all rated M = 3 or higher. Table 3A–SI shows the Mean and SD for each function.

### 3.3 Threats to trustworthy social robots

We explored whether participants consider various functions for which social robots are being deployed as potential threats to trustworthy social robots. Participants rated the perceived threat to six dimensions of trustworthy social robots (human autonomy, privacy, safety, fairness, diversity, and non-discrimination, societal well-being, and accountability) on a 5-point scale (1 = very low, 5 = very high) for each function discussed in the previous section.

The perceived threats were assessed using the median as a reference, with evaluations ranging from very low (Mdn = 1) to medium (Mdn = 3). None of the functions were rated above a median of 3 for any of the dimensions.

The functions perceived as more threatening were triage (i.e., social robots that measure vital signs to provide pre-diagnoses or prioritize patients according to the urgency of their symptoms) and banning entrance (i.e., social robots that prevent entry to a building for individuals who pose a public health threat, such as having a fever, or those who do not comply with rules, like wearing a mandatory face mask). For triage, threats to human autonomy, privacy, safety, and accountability were rated as medium (Mdn = 3). For banning entrance, threats to human autonomy, safety, fairness, diversity, and non-discrimination, and accountability were also rated as medium (Mdn = 3).

Following these, the functions of providing personalized information (i.e., social robots that inform patients about the characteristics of a medical condition, symptoms, and potential treatments) and monitoring (i.e., social robots that monitor patients in hospitals and alert medical staff to unusual situations) were next. For both personalized information and monitoring, threats to privacy, safety, and accountability were rated as medium (Mdn = 3). Additionally, for functions related to patient registration, data collection, patrolling, and home assistance, the threat to privacy was rated as medium (Mdn = 3).

Among the dimensions, privacy raised the most concern, being rated with a median of 3 on eight occasions. Safety and accountability were each rated with a median of 3 on four occasions. Human autonomy was rated with a median of 3 on two occasions, and fairness, diversity, and non-discrimination was rated with a median of 3 on one occasion. Societal well-being was not rated with a median of 3 or above on any occasion. Table 4A - SI shows the medians for each threat and function.

### 3.4 Care recipients

Participants evaluated on a 4-point scale (1 = no, definitely not - 4 = yes, definitely) whether European policymakers should support and promote the development and deployment of social robots to assist different groups of care recipients in the ideal European society that they envisioned for the near future. Robot assistance for the different groups were all rated very similarly, all yielding a median of 3 (yes, to some extent). Table 1 shows the mean and SD of the different care recipients that were rated by the participants.

**Table 1:** Mean and SD for care recipients.

Care recipients	M	SD
Conscious adult patients in a hospital	3.18	0.70
Unconscious adult patients in a hospital	2.8	0.85
Children patients in a hospital	2.81	0.84
Older adult patients in a hospital	3.09	0.76
Older adults with dementia in an elderly care center	2.88	0.88
Older adults with dementia in an occupational therapy center	2.87	0.87
Healthy older adults in an elderly care center	3.19	0.72
Healthy older adults living by themselves in their own homes	3.10	0.76
Children with autism in an occupational therapy center	2.79	0.88
Children with autism in their own homes	2.74	0.81

### 3.5 Vulnerability

We additionally examined whether perceived vulnerability was related to the willingness to promote social robots for different care recipients. Specifically, we assessed whether the more vulnerable a group of recipients was perceived to be, the less likely participants would accept robots taking care of that recipient.

Participants rated the perceived vulnerability of several recipient groups: conscious adult patients, unconscious adult patients, children patients, older adults with cognitive impairment, children with autism, and healthy older adults. For healthy older adults, we measured perceived vulnerability separately for two groups: those in elderly care and those at home by themselves. This was done to determine whether being alone (vs. surrounded by caregivers) influenced perceived vulnerability and the willingness to promote robot deployments depending on this circumstance. Table 2 shows the results.

**Table 2:** Mean and SD for perceived vulnerability.

Vulnerability	M	SD
Conscious adult patients	2.32	0.80
Unconscious adult patients	4.02	1.00
Children patients	3.82	0.92
Older adult patients	3.52	0.94
Older adults with dementia	3.83	0.93
Healthy older adults (elderly care)	2.71	0.83
Healthy older adults (by themselves at home)	2.85	1.04
Children with autism	3.85	0.93

We explored the correlations between perceived vulnerability and care recipients. We found an inverse correlation between willingness to promote social robots for the care recipient of a group and the perceived vulnerability of that group for most cases.

Willingness to promote social robots for conscious adult patients (r = −0.142, p < 0.01), unconscious adult patients (r = −0.106, p < 0.01), children patients (r = −0.172, p < 0.01), and older adult patients (r = −0.143, p < 0.01) in a hospital were all inversely correlated with the perceived vulnerability of the respective group.

Similarly, willingness to promote social robots for healthy older adults (r = −0.097, p < 0.01), and older adults with dementia (r = −0.073, p < 0.01) in an elderly care center were all inversely correlated with the perceived vulnerability of the respective group. Additionally, willingness to promote social robots for older adults with dementia in an occupational therapy center was inversely correlated with perceived vulnerability of that group (r = −0.083, p < 0.01). However, willingness to promote social robots for healthy older adults living at home by themselves was not significantly inversely correlated with perceived vulnerability of that group (r = −0.004, p = 0.88).

Finally, willingness to promote social robots for children with autism in an occupational therapy center (r = −0.180, p < 0.01), and for children with autism in their homes (r = −0.141, p < 0.01) were all inversely correlated with perceived vulnerability of the respective group.

### 3.6 Roles

We analyzed whether the role performed by the robot, as well as the degree of responsibility in that role (assisting a human vs. performing the role independently), influenced the willingness to promote social robots for healthcare. Participants answered whether, in the ideal European society they envisioned for the near future, European policymakers should support and promote the development and deployment of social robots performing various roles in the healthcare sector (referred to as acceptance).

Performing as a doctor and performing as a psychotherapist were the roles that faced the highest rejection, while assisting a receptionist and assisting a nurse received the highest acceptance. The degree of responsibility was a significant factor in acceptance. Assisting a human in roles such as nurse, doctor, receptionist, caregiver, or psychotherapist was consistently rated higher in acceptance compared to performing the role independently.

Specifically, acceptance for assisting a nurse in a hospital was significantly higher than for performing as a nurse (t(1091) = 45.938, p < 0.001). Similarly, acceptance for assisting a doctor in a hospital was significantly higher than for performing as a doctor (t(1091) = 50.507, p < 0.001). Acceptance for assisting a receptionist in a healthcare center was significantly higher than for performing as a receptionist (t(1091) = 30.805, p < 0.001). Similarity, acceptance for assisting a caregiver in an elderly care center was significantly higher than for performing as a caregiver in an elderly care center (t(1091) = 42.749, p < 0.001). In the same way, acceptance for assisting a caregiver in a private home was significantly higher than for performing as a caregiver in a private home (t(1091) = 38.351, p < 0.001). And finally, acceptance for assisting a psychotherapist in an occupational therapy center was significantly higher than for performing as a psychotherapist (t(1091) = 41.534, p < 0.001).

Table 3 shows the Means and SD for all roles. A Bonferroni correction was applied to all p-values due to multiple comparisons, adjusting the significance level to p < .0083.

**Table 3:** Mean and SD for the different roles potentially performed by the SARs.

Roles	M	SD
Assist a nurse in a hospital	3.36	0.63
Perform by themselves the tasks typically conducted by a nurse in a hospital	2.22	0.87
Assist a doctor in a hospital	3.11	0.72
Perform by themselves the tasks typically conducted by a doctor in a hospital	1.78	0.86
Assist a receptionist in a healthcare center	3.51	0.63
Perform by themselves the tasks typically conducted by a receptionist in a healthcare center	2.79	0.94
Assist a caregiver in an elderly care center	3.29	0.67
Perform by themselves the tasks typically conducted by a caregiver in an elderly care center	2.19	0.91
Assist a caregiver in a private home	3.25	0.69
Perform by themselves the tasks typically conducted by a caregiver in a private home	2.23	0.92
Assist a psychotherapist in an occupational therapy center	2.96	0.87
Perform by themselves the tasks typically conducted by a psychotherapist in an occupational therapy center	1.82	0.88

### 3.7 Attitudes towards robots by gender and age

We examined potential differences regarding attitudes towards robots by gender, as well as the relationship between attitudes towards robots and age. For attitudes towards robots, we created a scale from the following four items evaluating general attitudes towards robots: *robots are a good thing for society because they help people; robots steal people's jobs* (reversed); *robots are necessary as they can do jobs that are too hard or too dangerous for people;* and *the widespread use of robots can boost job opportunities*. The item *robots are a form of technology that requires careful management* was eliminated from the initial questionnaire based on a factorial analysis that did not support a simple factor structure (KMO = 0.65; Barlett's Test of Sphericity x^2^(10) = 664.823, p < 0.001). The final scale had a Cronbach's α of 0.65 (M = 2.96, SD = 0.48). We also used an item assessing the general view of robots (*Generally speaking, do you have a view of robots …*, rated from 1- very negative to 4 - very positive).

We found significant gender differences in attitudes towards robots. Males received higher median ranks than females on all three measures: general view of robots (Mann-Whitney U = 131501.000, Z = −2.993, p = 0.003), attitudes towards robots (Mann-Whitney U = 127337.500, Z = −3.159, p = 0.002), and attitudes towards robots for healthcare (Mann-Whitney U = 124436.000, Z = −3.750, p < 0.001).

No significant correlation was found between age and general attitudes towards robots (r = −0.027, p = 0.37) or with the general view of robots (r = −0.052, p = 0.08). However, we found a moderately significant inverse correlation between age and attitudes towards robots for healthcare (r = −0.088, p = 0.03), indicating that older adults showed a less positive attitude towards robots for healthcare.

### 3.8 Attitudes towards robots across European regions

For comparative analysis, the countries were grouped into three regions: Northern and Western Europe (Denmark, Sweden, Finland, Ireland, France, Germany, Netherlands, Austria, Belgium, Luxembourg), Southern Europe (Italy, Spain, Portugal, Greece, Cyprus), and Eastern Europe (Czechia, Hungary, Poland, Estonia, Latvia, Slovenia, Romania, Slovak Republic, Bulgaria, Croatia). This grouping was used to analyze cultural differences in attitudes towards robots, general view of robots, and attitude towards robots for healthcare. For these particular analyses, we included only the participants that had a residence and nationality from the same cluster (n total = 1049, Northern and Western n = 258, Southern = 512, Eastern = 279).

Kruskal-Wallis H tests were conducted to examine differences in attitudes towards robots across European regions.

Regarding attitudes towards robots, Eastern Europe had the highest mean rank (551.39), indicating the more positive attitudes, followed by Southern Europe (518.6). Western Europe had the lowest mean rank (509.16). However, there were no significant differences (H(2) = 3.131, p = 0.208).

Regarding general view of robots (H(2) = 8.056, p = 0.018), Eastern Europe had the highest mean rank (558.64), indicating a generally more positive view of robots compared to the other residence clusters. Southern Europe (513.03) and Western Europe (512.37) presented similar mean ranks, suggesting no major differences in general view of robots between these areas.

Regarding attitudes towards robots for healthcare (H(2) = 16.579, p < 0.001), Eastern Europe had the highest mean rank (586.42), indicating a generally more positive attitude towards robots for healthcare compared to the other regions. Southern Europe followed with a slightly lower mean rank (507.90), and Western Europe had the lowest mean rank (492.52), suggesting the least positive view on average.

### 3.9 Attitudes towards robots and previous experience with robots

Lastly, we explored the relationship between previous experience interacting with social robots and attitudes towards robots. For previous experience, we used the item: *Indicate your experience interacting with social robots* (1 - never; 3 - ample experience). For attitudes towards robots, we used the general attitudes towards robots scale and the item that assessed the general view of robots.

We found that previous experience interacting with social robots was positively correlated with the general view of robots (r = 0.121, p < 0.001), with attitudes towards robots, although this correlation did not reach significance (r = 0.084, p = 0.05), and with attitudes towards robots for healthcare (r = 0.121, p < 0.001), indicating that the more experience participants had interacting with robots, the more positive their views of robots were.

### 3.10 Attitudes towards robots and religious beliefs

We additionally examined the relationship between attitudes towards robots and religious beliefs, as well as between fear of robots and religious beliefs. For religious beliefs, we used the question: *To what level do you consider yourself to be religious?* (1 - not religious; 4 - very religious). For attitudes towards robots, we used the general attitudes towards robots scale (as described above) and the item that assessed the general view of robots. For fear of robots, we used the 12-item fear of robots scale.

We found a positive correlation between religious beliefs and fear of robots, indicating that the more religious participants were, the more fear they had of robots (r = 0.177, p < 0.01). We also found an inverse correlation between religious beliefs and the general view of robots, indicating that the more religious participants were, the less positive their views of robots (r = −0.062, p < 0.01). Additionally, we found an inverse correlation between religious beliefs and general attitudes towards robots, so that the more religious participants were, the less positive their attitudes towards robots (r = −0.131, p < 0.01). Religious beliefs was also negatively correlated with attitudes towards robots for healthcare, although not significant (r = −0.052, p = 0.08).

## 4 Discussion

Involving citizens in the decisions concerning SARs deployment in the EU is important to build a society that people find fair in terms of robot coexistence. To respond to this need, we conducted a large-scale survey among European citizens to explore their attitudes and opinions regarding the adoption of SARs for healthcare in the EU. Our study examined which functions citizens would support and which they consider a potential threat to the six dimensions of trustworthy SARs, namely, human autonomy, privacy, safety, societal well-being, accountability, and fairness, diversity, and non-discrimination. We additionally explored the relationships between the perceived vulnerability of the care recipient and acceptance, between attitudes towards robots and religious beliefs, between previous experience interacting with social robots and attitudes towards robots, and whether the degree of responsibility taken in performing a role affects acceptance.

Overall, the majority of the participants reported having a fairly positive attitude towards robots and robots for healthcare.

Concerning specific functions, the results of the study showed that the functions most negatively rated were related to triage and banning entrance to places. While further research is needed to investigate the underlying causes more in depth, these findings suggest that citizens might have particularly strong concerns over functions that involve giving robots authority to be able to make decisions over them. Giving robots the authority to make decisions about who gets medical attention first or who can enter a building might be seen as ethically problematic or they might distrust the ability of robots to make accurate and fair decisions in critical situations. Participants might feel that such decisions should remain under human control due to their complex and sensitive nature. Additionally, triage and banning entrance functions inherently involve processing sensitive personal health data. Participants might be concerned about the accuracy, security, and confidentiality of this information. The risk of data breaches or misuse of personal health information can evoke strong negative reactions. Overall, the general recommendation might be to avoid adopting SARs for functions that could make citizens uncomfortable, particularly those that require making critical decisions or handling sensitive personal data, as these could erode public trust in these type of robots.

Among the dimensions of trustworthy robots, a potential threat to privacy was the one that raised concern on more occasions. This observation is a critical insight for developers, indicating the necessity of prioritizing privacy safeguards in SARs. As robots become increasingly integrated into healthcare systems, developers should focus on implementing robust data encryption, secure data storage, and minimal data retention policies to ensure that personal information is protected against unauthorized access and breaches. Additionally, transparent privacy policies and the ability for users to control their own data can help mitigate privacy concerns and build trust between citizens and SARs.

In relation to gender and age, our results suggest that males, on average, expressed more positive views of robots compared to females. While age in general did not correlate with attitudes towards robots, older adults showed slightly more concern specifically about robots for healthcare. This concern might be linked to the increased perceived vulnerability of this group, aligning with the inverse correlation found between the perceived vulnerability of care recipients and acceptance. These findings highlight the importance of considering demographic variables when examining public perceptions of robots. Future research could explore gender and age as mediators to provide a more nuanced understanding of how these factors influence attitudes towards robots. This line of inquiry could offer deeper insights into the complex interplay between demographic characteristics and technology acceptance, ultimately enhancing our ability to design and implement robot technologies that are more broadly accepted across diverse populations. Additionally, we found a positive relationship between religious beliefs and fear of robots and a positive relationship between previous experience interacting with social robots and attitudes towards robots. This indicates that familiarity and hands-on experience with robots can significantly enhance acceptance and reduce fear. These insights emphasize the importance of exposure and education in shaping public attitudes towards robots, suggesting that increased interaction and familiarity with robots could mitigate fears and improve acceptance across different demographic groups. However, the correlation coefficients, although statistically significant in most cases, generally indicated weak associations when interpreted within the context of the effect sizes observed (e.g., = −0.142, <0.01 r = −0.142,p < 0.01 for conscious adult patients in a hospital, with only about 2.02% of the variance explained). Such results suggest that while there is a measurable relationship, the practical impact of perceived vulnerability on acceptance, religious beliefs on fear of robots, and previous experience on attitudes towards robots might be limited.

Also, the degree of responsibility in a role was a determining factor of acceptance so assisting a human nurse, doctor, receptionist, caregiver, or psychotherapist was better accepted than the robot performing the role by itself. These results are also indicative that citizens generally seem to prefer robots to function under human supervision as helpers, rather than autonomous decision-makers for performing critical roles in the healthcare system currently developed by humans. This preference also might suggest a broader societal inclination towards maintaining human oversight in roles that involve critical judgment, empathy, and ethical considerations. Thus, integrating robots under human supervision rather than as autonomous decision-makers for the roles we analyzed might positively contribute to social acceptance of SARs.

Regarding differences across regions, Eastern Europe had the more positive attitudes towards robots, followed by Southern Europe, and Northern-Western Europe had the least positive attitudes. While some of the tests yielded significant differences, these need to be interpreted with caution. We refrained from conducting post-hoc tests due to the limited sample size on each group and to avoid inflating the risk of Type I error, where we might mistakenly conclude significant differences exist.

Several limitations should be considered when interpreting the findings of this study. Firstly, while efforts were made to recruit a diverse sample, the majority of respondents were young adults aged 18–34, potentially limiting the generalizability of results across broader age demographics. Additionally, the study relied on digital survey distribution methods, which may have introduced bias towards individuals with higher digital literacy and accessibility. Furthermore, while the study aimed for representation across European nations, there was variability in response rates across countries, with larger numbers of participants from Italy, Portugal, and Poland, potentially skewing the overall national representation. Future research should aim for more balanced demographic representation and employ diverse recruitment strategies to enhance the inclusivity and applicability of findings across different demographic groups and national contexts.

We expect that the results of the survey will provide critical information that can be used to support citizen-centric policies that involve citizen's opinions regarding SAR coexistence. As a further line of work, we included in our survey a series of optional open-ended questions. In particular, a question regarding what aspects worried participants the most about social robots being implemented in society, a question asking whether there was any message the participants would like to send to the policymakers that will regulate social robot deployments in Europe, and a question for additional comments. We expect to be able to analyze the qualitative results of the survey to get more insights on the results in the future.

## CRediT authorship contribution statement

**Laura Aymerich-Franch:** Writing – review & editing, Writing – original draft, Visualization, Validation, Supervision, Software, Resources, Project administration, Methodology, Investigation, Funding acquisition, Formal analysis, Data curation, Conceptualization. **Emilia Gómez:** Writing – review & editing, Writing – original draft, Visualization, Validation, Supervision, Software, Resources, Project administration, Methodology, Investigation, Funding acquisition, Formal analysis, Data curation, Conceptualization.

## Declaration of competing interest

The authors declare that they have no known competing financial interests for the work reported in this paper.

## Data Availability

Data will be made available on request.
