# Supplementary material for: Public perception of socially assistive robots for healthcare in the EU: A large-scale survey
Source: Comput Hum Behav Rep. 2024 Aug;15:None. doi: 10.1016/j.chbr.2024.100465 (PMC11388560; doi:10.1016/j.chbr.2024.100465)
Supplement: Multimedia component 1 [file mmc1.docx]

**Supplementary Information:**

**Public perception of socially assistive robots for healthcare in the EU: a large-scale survey**

Complete list of functions evaluated by participants in the survey:

- Pick up and deliver objects in hospitals and elderly care centers
- Pick up and deliver objects circulating outdoors (e.g., in a residential area or a university campus)
- Telepresence, so that a patient in a hospital or a resident in an elderly care center can communicate with friends and relatives
- Monitor patients in hospitals and send alerts to the medical staff if an unusual situation is detected
- Register patients and book appointments at the reception of hospitals and medical centers
- Collect personal information of medical symptoms presented by patients on arrival at the hospital or medical center
- Provide general information in hospital aisles, such as giving locations or informing safety measures
- Provide personalized information such as informing about the characteristics of a particular medical condition, symptoms, and potential treatments to patients in hospitals
- Measure vital signs to inform medical personnel
- Measure vital signs so that the robots themselves can provide pre-diagnoses or do the triage (i.e., prioritize patients according to the degree of urgency) based on the symptoms
- Announce protocols and promote safety measures inside hospitals and medical centers, such as using sanitizer or face masks
- Advise to visit a doctor after pre-screening a person and detecting a fever and coughing
- Ban entrance to a building to people that present a threat to public health (e.g., have a fever) or do not comply with the rules (e.g., mandatory face mask)
- Patrol hospitals and nursing homes for security and alert the staff when a safety concern is detected
- Disinfect hospital buildings or nursing homes
- Entertain patients in a hospital or residents in a nursing home with activities such as singing, games, jokes, or dancing
- Offer edutainment activities to older adults such as playing memory games
- Offer motivational conversation to patients of a hospital
- Express and interpret emotions to communicate
- Provide touch interaction for emotional support
- Co-habit with older adults at their home for assistance
- Remind older adults living by themselves to take a medicine
- Instruct physical exercise, relaxation techniques, dancing, and yoga in hospitals and nursing homes
- Support therapy for autism or dementia
- Translate conversations between doctors and foreign patients in a hospital
- Used as patient simulators for skills training of medical students

Complete list of questions included in the questionnaire:

**QUESTIONNAIRE**

**I. DEMOGRAPHICS**

1. Nationality (check all that apply)
2. Country of residence (check all that apply)
3. Gender (male, female, other)
4. Age
5. Current Occupation

- Student
- Self-employed
- Employed
- Not-working
- Retired
- Other

6. Have you ever worked in the area of healthcare?

- I work in the area of healthcare at present
- I worked in the area of healthcare in the past, but not anymore
- I have never worked in the area of healthcare

7. Do you live in a…

- Rural area or village
- Small or middle sized town
- Large town or city

8. Please, indicate your level of studies

- Less than a high school diploma
- High school degree or equivalent
- Some college, no degree
- Bachelor’s degree or equivalent
- Master’s degree or equivalent
- Professional degree
- Doctorate

9. What describes best your current marital status?

- Single
- Married, in a domestic partnership, with a partner
- Widowed
- Divorced or Separated
- Other

10. What describes best your current household composition?

- Living by myself
- Living with partner
- Living with partner and child(ren)
- Living with child(ren)
- Living with parent(s)
- Living with housemate(s)
- Other

11. During the last twelve months, would you say you had difficulties to pay your bills at the end of the month…?

- Most of the time
- From time to time
- Almost never
- Never

12. Do you see yourself and your household belonging to…?

- The lower class of society
- The lower middle class of society
- The middle class of society
- The upper middle class of society
- The higher class of society

13. To what level, do you consider yourself to be religious?

- Not religious
- Slightly religious
- Moderately religious
- Very religious

14. What religious family do you belong to or identify yourself most close to?

- Christian
- Muslim
- Jewish
- Buddhist
- Hindu
- Other
- None

**II. RELATIONSHIP WITH ROBOTS & TECHNOLOGY**

1. Do you have a background in Computer Science, Engineering, or Robotics?

- Yes
- No

2. Please indicate your level of programming skills

- None
- Basic
- Medium
- Advanced

3. Indicate your general level of interest in scientific discoveries and technological developments

- Not interested
- Moderately interested
- Very interested

4. ​​Have you ever used, or are you currently using robots...

At home (e.g. a robotic vacuum cleaner)

- - Yes
  - No

At work (e.g. industrial robot)

- - Yes
  - No

Other places (e.g., shopping mall, airport…)

- - Yes
  - No

5.Please indicate your experience interacting with social robots.

- I have never interacted with social robots
- I have occasionally interacted with social robots
- I have ample experience interacting with social robots

6. Generally speaking, do you have a view of robots…

- Very negative
- Fairly negative
- Fairly positive
- Very positive

7. To what extent you agree or disagree with the following statements about robots

*(1 - totally disagree, 2- somewhat disagree, 2 - somewhat agree, 4 - totally agree):*

- Robots are a good thing for society, because they help people
- Robots steal peoples’ jobs
- Robots are necessary as they can do jobs that are too hard or too dangerous for people
- Robots are a form of technology that requires careful management
- Widespread use of robots can boost job opportunities in the EU

8. To what extent you agree or disagree with the following statements about robots for healthcare

*(1 - totally disagree, 2- somewhat disagree, 2 - somewhat agree, 4 - totally agree):*

- Robots for healthcare should be promoted in the EU
- Robots for healthcare should be banned in the EU
- Robots for healthcare can be beneficial for the EU economy
- Robots for healthcare can be beneficial for the EU citizens

9. How afraid are you of the following:

*(1 - not at all, 2 - slightly, 3 - moderately, 4 - very, 5- extremely)*

- Robots that can make their own decisions and take their own actions
- Robots replacing people in the workforce
- Robots becoming more intelligent than humans
- Robots enslaving humans
- Robots manipulating humans
- Robots destroying humanity
- Robots hurting people
- Robots in general
- Humans trusting robots to do work
- Humans receiving divine punishment for creating robots that resemble a human
- Humans establishing emotional links with robots
- Humans having sex with robots

**III. FUNCTIONS**

1. In the ideal European society that you envision for the near future, European policymakers support and promote the development and deployment of social robots that perform the following functions:

*(1 = totally disagree - 2 = somewhat disagree - 3 = somewhat agree - 4 = totally agree)*

- Pick up and deliver objects in hospitals and elderly care centers
- Pick up and deliver objects circulating outdoors (e.g., in a residential area or a university campus)
- Telepresence, so that a patient in a hospital or a resident in an elderly care center can communicate with friends and relatives
- Monitor patients in hospitals and send alerts to the medical staff if an unusual situation is detected
- Register patients and book appointments at the reception of hospitals and medical centers
- Collect personal information of medical symptoms presented by patients on arrival at the hospital or medical center
- Provide general information in hospital aisles, such as giving locations or informing safety measures
- Provide personalized information such as informing about the characteristics of a particular medical condition, symptoms, and potential treatments to patients in hospitals
- Measure vital signs to inform medical personnel
- Measure vital signs so that the robots themselves can provide pre-diagnoses or do the triage (i.e., prioritize patients according to the degree of urgency) based on the symptoms
- Announce protocols and promote safety measures inside hospitals and medical centers, such as using sanitizer or face masks
- Advise to visit a doctor after pre-screening a person and detecting a fever and coughing
- Ban entrance to a building to people that present a threat to public health (e.g., have a fever) or do not comply with the rules (e.g., mandatory face mask)
- Patrol hospitals and nursing homes for security and alert the staff when a safety concern is detected
- Disinfect hospital buildings or nursing homes
- Entertain patients in a hospital or residents in a nursing home with activities such as singing, games, jokes, or dancing
- Offer edutainment activities to older adults such as playing memory games
- Offer motivational conversation to patients of a hospital
- Express and interpret emotions to communicate
- Provide touch interaction for emotional support
- Co-habit with older adults at their home for assistance
- Remind older adults living by themselves to take a medicine
- Instruct physical exercise, relaxation techniques, dancing, and yoga in hospitals and nursing homes
- Support therapy for autism or dementia
- Translate conversations between doctors and foreign patients in a hospital
- Used as patient simulators for skills training of medical students

2. In your opinion, social robots that [same list of functions than in the previous question] might entail a (low / high) risk to…

*(1 - Very low, 2 - Low, 3 - Medium, 4 - High, 5- Very high)*

- Human Autonomy
- Privacy
- Safety
- Fairness, diversity, and non-discrimination
- Societal well-being
- Accountability

**IV. ADOPTION**

In the ideal European society that you envision for the near future, European policymakers support and promote the development and deployment of social robots that assist the following persons:

*(1 = no, definitely not, 2 - leaning towards no, 3 - yes, to some extent, - 4 = yes, definitely)*

- conscious adult patients in a hospital
- unconscious adult patients in a hospital
- children patients in a hospital
- older adult patients in a hospital
- healthy older adults in an elderly care center
- older adults with dementia in an elderly care center
- children with autism in an occupational therapy center
- older adults with dementia in an occupational therapy center
- healthy older adults living alone in their own homes
- children with autism in their own homes

**V. PERCEIVED VULNERABILITY**

In your opinion, how vulnerable are the following individuals, on average…

*(1 - not at all vulnerable, 5 - extremely vulnerable)*

- Adult patients in a hospital
- Unconscious adult patients in a hospital
- Children patients in a hospital
- Older adult patients in a hospital
- Healthy older adults living in a nursing home
- Healthy older adults living alone at home
- Older adults with cognitive impairment in a nursing home
- Children with autism

**VI. ROLES**

In the ideal European society that you envision for the near future, European policymakers support and promote the development and deployment of social robots that perform the following roles:

*(1 = no, definitely not, 2 - leaning towards no, 3 - yes, to some extent, - 4 = yes, definitely)*

- assist a nurse in a hospital
- perform by themselves the tasks typically conducted by a nurse in a hospital
- assist a doctor in a hospital perform by themselves the tasks typically conducted by a doctor in a hospital
- assist a receptionist in a healthcare center perform by themselves the tasks typically conducted by a receptionist in a healthcare center
- assist a caregiver in an elderly care center perform by themselves the tasks typically conducted
- by a caregiver in an elderly care center
- assist a caregiver in a private home perform by themselves the tasks typically conducted by a caregiver in a private home assist a psychotherapist in an occupational therapy
- center perform by themselves the tasks typically conducted by a psychotherapist in an occupational therapy center

Table 1A. Nationality of participants

| **Nationality** | **Total** |
| --- | --- |
| Austria | 18 |
| Belgium | 22 |
| Bulgaria | 2 |
| Croatia | 4 |
| Cyprus | 1 |
| Czechia | 22 |
| Denmark | 1 |
| Estonia | 17 |
| Finland | 17 |
| France | 53 |
| Germany | 73 |
| Greece | 88 |
| Hungary | 54 |
| Ireland | 45 |
| Italy | 163 |
| Latvia | 16 |
| Luxembourg | 2 |
| Netherlands | 24 |
| Other | 2 |
| Poland | 152 |
| Portugal | 192 |
| Romania | 6 |
| Slovak Republic | 1 |
| Slovenia | 21 |
| Spain | 87 |
| Sweden | 9 |
| Total | 1092 |

Table 2A. Country of residence of participants

| **Country of residence** | **Total** |
| --- | --- |
| Austria | 26 |
| Belgium | 23 |
| Czechia | 24 |
| Denmark | 3 |
| Estonia | 16 |
| Finland | 17 |
| France | 55 |
| Germany | 72 |
| Greece | 81 |
| Hungary | 54 |
| Ireland | 49 |
| Italy | 159 |
| Latvia | 15 |
| Luxembourg | 2 |
| Netherlands | 32 |
| Poland | 150 |
| Portugal | 188 |
| Slovenia | 22 |
| Spain | 93 |
| Sweden | 11 |
| Total | 1092 |

Table 3A. Means and SD by function

| **Function** | **M** | **SD** |
| --- | --- | --- |
| Pick up and deliver objects in hospitals and elderly care centers | 3.53 | .65 |
| Pick up and deliver objects circulating outdoors (e.g., in a residential area or a university campus) | 3.45 | .69 |
| Telepresence, so that a patient in a hospital or a resident in an elderly care center can communicate with friends and relatives | 3.54 | .66 |
| Monitor patients in hospitals and send alerts to the medical staff if an unusual situation is detected | 3.56 | .66 |
| Register patients and book appointments at the reception of hospitals and medical centers | 3.08 | .86 |
| **Collect personal information of medical symptoms presented by patients on arrival at the hospital or medical center** | **2.75** | **.90** |
| Provide general information in hospital aisles, such as giving locations or informing safety measures | 3.52 | .70 |
| **Provide personalized information such as informing about the characteristics of a particular medical condition, symptoms, and potential treatments to patients in hospitals** | **2.71** | **.93** |
| Measure vital signs to inform medical personnel | 3.29 | .81 |
| **Measure vital signs so that the robots themselves can provide pre-diagnoses or do the triage (i.e., prioritize patients according to the degree of urgency) based on the symptoms** | **2.42** | **.94** |
| Announce protocols and promote safety measures inside hospitals and medical centers, such as using sanitizer or face masks | 3.43 | .76 |
| Advise to visit a doctor after pre-screening a person and detecting a fever and coughing | 3.09 | .85 |
| **Ban entrance to a building to people that present a threat to public health (e.g., have a fever) or do not comply with the rules (e.g., mandatory face mask)** | **2.48** | **.97** |
| Patrol hospitals and nursing homes for security and alert the staff when a safety concern is detected | 3 | .90 |
| Disinfect hospital buildings or nursing homes | 3.58 | .66 |
| Entertain patients in a hospital or residents in a nursing home with activities such as singing, games, jokes, or dancing | 3.12 | .88 |
| Offer edutainment activities to older adults such as playing memory games | 3.4 | .76 |
| **Offer motivational conversation to patients of a hospital** | **2.8** | **.97** |
| **Express and interpret emotions to communicate** | **2.63** | **.92** |
| **Provide touch interaction for emotional support** | **2.55** | **.95** |
| Co-habit with older adults at their home for assistance | 3.09 | .82 |
| Remind older adults living by themselves to take a medicine | 3.6 | .62 |
| Instruct physical exercise, relaxation techniques, dancing, and yoga in hospitals and nursing homes | 3.15 | .87 |
| Support therapy for autism or dementia | 3.1 | .89 |
| Translate conversations between doctors and foreign patients in a hospital | 3.54 | .70 |
| Used as patient simulators for skills training of medical students | 3.44 | .73 |

Table 4A. Medians and IQ for each threat and function

| **Threats to trustworthy social robots** | **Mdn** | **IQR** |
| --- | --- | --- |
| Delivery indoors: Human Autonomy | 2 | 2 |
| Delivery indoors: Privacy | 2 | 2 |
| Delivery indoors: Safety | 2 | 2 |
| Delivery indoors: Fairness, diversity, and non-discrimination | 1 | 1 |
| Delivery indoors: Societal well-being | 1 | 1 |
| Delivery indoors: Accountability | 2 | 2 |
| Delivery outdoors: Human Autonomy | 2 | 1 |
| Delivery outdoors: Privacy | 2 | 1 |
| Delivery outdoors: Safety | 2 | 2 |
| Delivery outdoors: Fairness, diversity, and non-discrimination | 1 | 1 |
| Delivery outdoors: Societal well-being | 1 | 1 |
| Delivery outdoors: Accountability | 2 | 2 |
| Telepresence: Human Autonomy | 2 | 2 |
| **Telepresence: Privacy** | **3** | **2** |
| Telepresence: Safety | 2 | 1 |
| Telepresence: Fairness, diversity, and non-discrimination | 1 | 1 |
| Telepresence: Societal well-being | 1 | 1 |
| Telepresence: Accountability | 2 | 1 |
| Registration: Human Autonomy | 2 | 2 |
| **Registration: Privacy** | **3** | **2** |
| Registration: Safety | 2 | 1 |
| Registration: Fairness, diversity, and non-discrimination | 1 | 1 |
| Registration: Societal well-being | 2 | 2 |
| Registration: Accountability | 2 | 2 |
| Data collection: Human Autonomy | 2 | 2 |
| **Data collection: Privacy** | **3** | **2** |
| Data collection: Safety | 2 | 2 |
| Data collection: Fairness, diversity, and non-discrimination | 2 | 2 |
| Data collection: Societal well-being | 2 | 2 |
| Data collection: Accountability | 2 | 2 |
| General information: Human Autonomy | 1 | 1 |
| General information: Privacy | 1 | 1 |
| General information: Safety | 1 | 1 |
| General information: Fairness, diversity, and non-discrimination | 1 | 1 |
| General information: Societal well-being | 1 | 1 |
| General information: Accountability | 1 | 1 |
| Personal information: Human Autonomy | 2 | 2 |
| **Personal information: Privacy** | **3** | **2** |
| **Personal information: Safety** | **3** | **1** |
| Personal information: Fairness, diversity, and non-discrimination | 1 | 1 |
| Personal information: Societal well-being | 2 | 2 |
| **Personal information: Accountability** | **3** | **3** |
| Monitoring: Human Autonomy | 2 | 2 |
| **Monitoring: Privacy** | **3** | **1** |
| **Monitoring: Safety** | **3** | **3** |
| Monitoring: Fairness, diversity, and non-discrimination | 1 | 1 |
| Monitoring: Societal well-being | 2 | 2 |
| **Monitoring: Accountability** | **3** | **3** |
| Vital signs: Human Autonomy | 2 | 2 |
| Vital signs: Privacy | 2 | 2 |
| Vital signs: Safety | 2 | 3 |
| Vital signs: Fairness, diversity, and non-discrimination | 1 | 1 |
| Vital signs: Societal well-being | 1 | 1 |
| Vital signs: Accountability | 2 | 2 |
| **Triage: Human Autonomy** | **3** | **2** |
| **Triage: Privacy** | **3** | **2** |
| **Triage: Safety** | **3** | **2** |
| Triage: Fairness, diversity, and non-discrimination | 2 | 2 |
| Triage: Societal well-being | 2 | 2 |
| **Triage: Accountability** | **3** | **2** |
| Protocols: Human Autonomy | 1 | 1 |
| Protocols: Privacy | 1 | 1 |
| Protocols: Safety | 1 | 1 |
| Protocols: Fairness, diversity, and non-discrimination | 1 | 1 |
| Protocols: Societal well-being | 1 | 1 |
| Protocols: Accountability | 1 | 1 |
| Advise: Human Autonomy | 2 | 2 |
| Advise: Privacy | 2 | 2 |
| Advise: Safety | 2 | 2 |
| Advise: Fairness, diversity, and non-discrimination | 1 | 1 |
| Advise: Societal well-being | 1 | 2 |
| Advise: Accountability | 2 | 2 |
| **Ban entrance: Human Autonomy** | **3** | **2** |
| Ban entrance: Privacy | 2 | 3 |
| **Ban entrance: Safety** | **3** | **2** |
| **Ban entrance: Fairness, diversity, and non-discrimination** | **3** | **3** |
| Ban entrance: Societal well-being | 2 | 2 |
| **Ban entrance: Accountability** | **3** | **2** |
| Patrolling: Human Autonomy | 2 | 2 |
| **Patrolling: Privacy** | **3** | **1** |
| Patrolling: Safety | 2 | 2 |
| Patrolling: Fairness, diversity, and non-discrimination | 2 | 2 |
| Patrolling: Societal well-being | 2 | 2 |
| Patrolling: Accountability | 2 | 2 |
| Disinfection: Human Autonomy | 1 | 1 |
| Disinfection: Privacy | 1 | 1 |
| Disinfection: Safety | 2 | 2 |
| Disinfection: Fairness, diversity, and non-discrimination | 1 | 1 |
| Disinfection: Societal well-being | 1 | 1 |
| Disinfection: Accountability | 1 | 2 |
| Entertainment: Human Autonomy | 2 | 2 |
| Entertainment: Privacy | 2 | 1 |
| Entertainment: Safety | 1 | 1 |
| Entertainment: Fairness, diversity, and non-discrimination | 1 | 1 |
| Entertainment: Societal well-being | 1 | 2 |
| Entertainment: Accountability | 1 | 1 |
| Edutainment: Human Autonomy | 2 | 2 |
| Edutainment: Privacy | 1 | 1 |
| Edutainment: Safety | 1 | 1 |
| Edutainment: Fairness, diversity, and non-discrimination | 1 | 1 |
| Edutainment: Societal well-being | 1 | 1 |
| Edutainment: Accountability | 1 | 1 |
| Motivation: Human Autonomy | 2 | 2 |
| Motivation: Privacy | 2 | 2 |
| Motivation: Safety | 1 | 2 |
| Motivation: Fairness, diversity, and non-discrimination | 1 | 1 |
| Motivation: Societal well-being | 2 | 2 |
| Motivation: Accountability | 2 | 2 |
| Emotions: Human Autonomy | 2 | 2 |
| Emotions: Privacy | 2 | 2 |
| Emotions: Safety | 2 | 2 |
| Emotions: Fairness, diversity, and non-discrimination | 2 | 2 |
| Emotions: Societal well-being | 2 | 2 |
| Emotions: Accountability | 2 | 2 |
| Touch: Human Autonomy | 2 | 2 |
| Touch: Privacy | 2 | 2 |
| Touch: Safety | 2 | 2 |
| Touch: Fairness, diversity, and non-discrimination | 1 | 1 |
| Touch: Societal well-being | 2 | 2 |
| Touch: Accountability | 2 | 2 |
| Home assistance: Human Autonomy | 2 | 1 |
| **Home assistance: Privacy** | **3** | **2** |
| Home assistance: Safety | 2 | 1 |
| Home assistance: Fairness, diversity, and non-discrimination | 1 | 1 |
| Home assistance: Societal well-being | 2 | 2 |
| Home assistance: Accountability | 2 | 2 |
| Medication: Human Autonomy | 2 | 2 |
| Medication: Privacy | 2 | 2 |
| Medication: Safety | 2 | 2 |
| Medication: Fairness, diversity, and non-discrimination | 1 | 1 |
| Medication: Societal well-being | 1 | 1 |
| Medication: Accountability | 2 | 2 |
| Exercise: Human Autonomy | 2 | 2 |
| Exercise: Privacy | 1 | 1 |
| Exercise: Safety | 2 | 2 |
| Exercise: Fairness, diversity, and non-discrimination | 1 | 1 |
| Exercise: Societal well-being | 1 | 1 |
| Exercise: Accountability | 2 | 2 |
| Therapy: Human Autonomy | 2 | 2 |
| Therapy: Privacy | 2 | 2 |
| Therapy: Safety | 2 | 2 |
| Therapy: Fairness, diversity, and non-discrimination | 1 | 1 |
| Therapy: Societal well-being | 2 | 2 |
| Therapy: Accountability | 2 | 2 |
| Translation: Human Autonomy | 2 | 2 |
| Translation: Privacy | 2 | 2 |
| Translation: Safety | 2 | 2 |
| Translation: Fairness, diversity, and non-discrimination | 1 | 1 |
| Translation: Societal well-being | 1 | 1 |
| Translation: Accountability | 2 | 2 |
| Simulation: Human Autonomy | 1 | 1 |
| Simulation: Privacy | 1 | 1 |
| Simulation: Safety | 1 | 2 |
| Simulation: Fairness, diversity, and non-discrimination | 1 | 1 |
| Simulation: Societal well-being | 1 | 1 |
| Simulation: Accountability | 1 | 2 |

*Note.* Combinations of function x trustworthy dimension with the highest medians (= 3) are in bold
